# LCZ696 mitigates diabetic-induced nephropathy through inhibiting oxidative stress, NF-κB mediated inflammation and glomerulosclerosis in rats

**DOI:** 10.7717/peerj.9196

**Published:** 2020-06-19

**Authors:** Mohamed Mohany, Ahmed Z. Alanazi, Faleh Alqahtani, Osamah M. Belali, Mohammed M. Ahmed, Salim S. Al-Rejaie

**Affiliations:** Department of Pharmacology and Toxicology, College of Pharmacy, King Saud University, Riyadh, Saudi Arabia

**Keywords:** Diabetic nephropathy, LCZ696, Oxidative stress, Inflammation, Glomerulosclerosis

## Abstract

**Background:**

Diabetic nephropathy (DN) is among the most common microvascular complications of diabetes resulting in end-stage renal disease and therefore search for candidates which can ameliorate the kidney function is needed simultaneously with standard diabetic pharmacotherapy. The current study was aimed to investigate the effect of long term sacubitril/valsartan therapy (LCZ696) in diabetic rats to assess its ameliorative impact against various pathological parameters such as oxidative stress, inflammation and glomerulosclerosis associated with chronic DN.

**Methods:**

A single dose (60 mg/kg/day) of STZ was used to induce type 1 diabetes in adult male wistar rats. 2 weeks after diabetes induction, these rats were treated orally with valsartan (31 mg/kg) or LCZ696 (68 mg/kg) for 6 weeks. At end of the treatment period, serum and kidney samples were collected and analyzed. The serum levels of glucose, insulin, urea, creatinine, TNF-α, IL-1β, IL-6 and IL-10 levels were estimated. In renal tissue homogenate, the levels of inflammatory markers such as TNF-α, IL-1β, IL-6, NF-kB along with oxidative stress biomarkers including thiobarbituric acid-reacting substances (TBARs), glutathione (GSH), superoxide dismutase (SOD), catalase (CAT), glutathione peroxidase (GPx), glutathione S-transferase (GST) were assessed. Histological changes were observed in kidney.

**Results:**

Time course therapy with****LCZ696 and valsartan in diabetic rats resulted in significant reduction of serum glucose, urea and creatinine levels (*P* < 0.05). Additionally, serum of treated diabetic rats showed a diminution in inflammatory (TNF-α, IL-1β, IL-6) and increment in anti-inflammatory (IL-10) cytokines levels (*P* < 0.05). Tissue homogenate of the kidney extracted from LCZ696 and valsartan treated diabetic rats revealed a substantial reduction in the levels of inflammatory markers such as TNF-α, IL-1β, IL-6, NF-kB and sufficient restoration of anti-oxidant enzyme levels (*P* < 0.05). Finally, in the histological sections of the kidney, prevention of renal injury was observed with limited necrosis and inflammatory cells infiltration.

**Conclusion:**

Present data suggest that LCZ696 has sufficient therapeutic potential to restrict DN progression through inhibiting inflammation, oxidative stress and glomerulosclerosis.

## Introduction

Diabetes mellitus (DM) is the most common metabolic disorder that negatively affects all aspects of body metabolism, contributing to multiple complications and dysfunction of the tissues (Carlsson, Andersson & Ahlbom, 2016; Sharma, Nazareth & Petersen, 2016). Diabetic nephropathy (DN) is the serious complication among people associated with DM and accounts 50% of end-stage renal diseases worldwide (Kanwar et al., 2011; Sulaiman, 2019). Notably, unremitting hyperglycemia is one of the primary causes of DN, which can ultimately result in damage to blood vessels, chronic inflammation and renal injury (Duran-Salgado & Rubio-Guerra, 2014). Generally, nephropathy is a common cause of mortality in patients with DM (Skupien et al., 2019). This syndrome is characterized by the progressive loss of renal functions and persistent albuminuria (Lim, 2014) followed by certain pathological changes such as glomerular mesangial expansion and accumulation of extracellular matrix as indicators of the incidence of glomerulosclerosis (Peng et al., 2019). Moreover, high levels of glucose can impair the growth of cells overtime and the production of growth factors as well as the occurrence of genes that eventually increase the extracellular matrix (Vallon & Komers, 2011). DN has several serious consequences such as fluid retention, imbalanced electrolytes and blood vessels damages. Therefore, DN is considered the causative factor for many health problems including hypertension, heart and blood vessels diseases (Petrie, Guzik & Touyz, 2018).

A large body of evidence indicates that inflammatory and oxidative stress signaling pathways are involved in the pathogenesis of DN (Hameed et al., 2018; Kato & Natarajan, 2014). Hence, the control of DN could be accomplished by targeting these two pathways. Oxidative stress is induced by an imbalance between the production of free radicals and the protection of antioxidants in cells that can cause tissue damage through necrosis and apoptosis (Pizzino et al., 2017). The most devastating effect of oxidative stress is the observed damage in proteins, lipids and DNA (Liguori et al., 2018). In addition, several studies have demonstrated an elevation of pro-inflammatory cytokines such as tumor necrosis factor-α (TNF-α), interleukin-1β (IL-1β) in DN patients (Chang et al., 2016) and in experimental animal models as well (Yang, 2019). In addition to renal inflammation, this increase in inflammatory factors disturb the systemic immune functions. Furthermore, uncontrolled hyperglycemia and oxidative stress have been shown to trigger transcription factors such as the nuclear factor kappa B (NF-κB) which enhance apoptotic, fibrotic and inflammatory processes which play a major role in cell injury and other complications (Singh et al., 2014). Thus the NF-κB is considered a potential target in the management of vascular complications of diabetes (Suryavanshi & Kulkarni, 2017).

The renin-angiotensin system (RAS) plays a key and regulatory role in most mechanisms involved in progression of renal diseases particularly DN (Yang & Xu, 2017). The inhibition of angiotensin II (Ang II) function using either angiotensin-converting enzyme inhibitors (ACEIs) or Ang II receptor blockers (ARBs) is important therapy for DN management (Baltatzi, Savopoulos & Hatzitolios, 2011). Valsartan (angiotensin II receptor blocker) has been found to reduce podocyte injury, oxidative stress and inflammation in a mouse model of DN (Zhou et al., 2014). LCZ696 belongs to a new class of drug called angiotensin receptor-neprilysin inhibitors (ARNIs) which combines an antagonist of neprilysin with ARB in a definite proportion. The clinical trials have shown that after LCZ696 treatment, significant decrease in the overall mortality and heart failure (HF) was noticed in hospitalizations (Jessup et al., 2014). Recently, experimental studies showed that LCZ696 was therapeutically superior to valsartan in modulation of RAS in spontaneously hypertensive female rats (Zhao et al., 2019). Additionally, it has been demonstrated that LCZ696 therapy delays the progression of chronic kidney disease in animal model (Jing et al., 2017). Accordingly, the present study was designed to evaluate the impact of LCZ696 administration on renal functions, oxidative stress, inflammation and glomerulosclerosis in streptozotocin induced diabetic rat model.

## Material and Methods

### Animal handling

Adult male Wistar rats weighing 260–300 g were obtained from the Central Animal Facility, Pharmacy College, King Saud University, where they were maintained and monitored in a specific pathogen-free environment. All experimental procedures including euthanasia were conducted in accordance with the National Institute of Health Guide for the Care and Use of Laboratory Animals, Institute for Laboratory Animal Research (NIH Publications No. 80-23; 1996) as well as The King Saud University Research Ethics Committee (approval number SE-19-118). All animals were allowed to acclimatize in polycarbonate cages inside a well-ventilated room for 7 days prior to experimentation. The animals were maintained under standard laboratory conditions (temperature of 23 °C, relative humidity of 60–70%, and 12-h light/dark cycle). The diet content protein 20%, fat 4%, fiber 3.5%, ash 6% and with the total energy 2850 Kcal/kg was purchased from the Grain Silos & Flour Mills Organization (GSFMO), Riyadh, Saudi Arabia. The animals were provided with food and water ad libitum.

### Diabetic induction

Diabetes mellitus was induced by a single intraperitoneal injection (60 mg/kg) of streptozotocin  (STZ) in overnight fasted rats by dissolving it in freshly prepared 0.1 M citrate buffer, pH 4.5 as previously described (Alotaibi et al., 2019). After STZ injection, the rats received free access of dextrose solution (5%) for 24 h to avoid hypoglycemic shock. Two-days after the STZ injection, blood sugar levels were tested (mg/dl) by using strips on glucometer (ACCU-CHEK ACTIVE, Roche, Germany). Animals with fasting blood glucose levels >110 mg/dl were considered as type 1 diabetic rats (Ojiako, Chikezie & Ogbuji, 2015) and included in the study.

### Experimental design

Eighteen diabetic male rats were randomly divided into three groups with six rats in each group and same number of normal rats were divided into other three groups.

1. Normal rats were treated with vehicle (NC).
2. Diabetic rats treated with vehicle (DC).
3. Normal rats were treated with valsartan (31 mg/kg/day) orally (NV).
4. Normal rats were treated with LCZ696 (68 mg/kg/day) orally (NSV).
5. Diabetic rats were treated with valsartan (31 mg/kg/day) orally (DV).
6. Diabetic rats were treated with LCZ696 (68 mg/kg/day) orally (DSV).

Valsartan (Tabuvan^®^) and LCZ696 (EntrestoTM) tablets were suspended in 0.5% carboxymethyl cellulose (CMC) and administered via oral gavage in a volume of 0.5 ml/100 g body weight of each animal. Doses for both the drugs were selected from the literature (Habibi et al., 2019). The control and STZ groups received similar volumes of 0.5% CMC during the experiment period. Treatment started two weeks after the diabetic induction and continued for six weeks.

### Samples collection

After the treatment period of 6 weeks, the animals were overnight fasted and were anesthetized with ketamine (Hikma Pharmaceuticals, Jordan, 94 mg/kg)/xylazine (Laboratories Calier, Spain, 10 mg/kg) mixture. Blood samples were withdrawn from the heart and placed into clean tubes, then the serum samples were separated by centrifugation at 3,000 rpm (800 g) for 10 min and stored at −80 °C until analysis. Both the kidneys were dissected, and small portion of kidney from each animal was immersed and fixed in 10% neutral buffer formalin (pH 7.4) for subsequent use in histopathological examinations. The other part of kidney samples immediately immersed in liquid nitrogen for a minute and then stored at −80 °C until analysis.

### Serum analysis

Serum levels of glucose and insulin were assessed by a commercially available kit (RANDOX Laboratories Ltd., UK and SPI bio, France, Millipore, EZRMI-13K, respectively). Serum creatinine and urea levels were measured by colorimetric methods (Linear Chemicals, Barcelona, Spain). Serum levels of IL-1β, TNF-α, IL-6 and IL-10 were determined by following the ELISA techniques (Thermo Scientific, Rockford, IL, USA).

### Tissue analysis

Small portions of kidneys were homogenized in physiological buffer (1:10, w/v). In order to remove cell debris, this homogenate was centrifuged at 1,000 rpm for 10 min at 4 °C. After discarding the pellets and to obtain post-mitochondrial supernatant, a portion of supernatant was centrifuged again at 12,000 rpm for 20 min and total protein concentrations in kidneys were measured according to Lowry assay (1951) (Lowry et al., 1951) using bovine serum albumin as a standard. Thiobarbituric acid reactive substances (TBARS) and glutathione (GSH) levels were measured by using ELISA kits (Cayman Chemical Co., USA). Renal levels of IL-1β, TNF-α, IL-6 and NF-κB were determined by following the ELISA techniques (Thermo Scientific, Rockford, IL, USA). In post-mitochondria supernatants of kidney samples, enzymatic activities of superoxide dismutase (SOD), catalase (CAT), glutathione peroxidase (GPx) and glutathione-S-transferase (GST) were measured by using ELISA kits (R&D systems Inc., USA).

### Renal histological evaluation

Rat kidneys were fixed in 10% formalin, dehydrated, and then embedded with paraffin wax. Using an automated microtome (Leica RM 2125 RM, Leica Microsystems, Nussloch, Germany), sections (5–7 µm) were obtained and mounted on glass slides. All slides were stained with hematoxylin and eosin (H&E) and examined using a light microscope Nikon Eclipse E600 with a digital high-resolution camera by an experienced pathologist in a blinded manner.

### Evaluation of leukocyte infiltration

Leukocyte infiltration was semi-quantitatively assessed by an experienced pathologist blinded to the study groups according to the guidelines described by Velasquez et al. (1997). The intensity of leukocyte infiltration on H&E stained renal cortex sections was obtained by assigning a score of 1–4 to each glomerulous as follows: normal = 0; up to 25% involvement = 1; 25%–50% involvement = 2; 50%–75% involvement = 3 and more than 75% involvement = 4.

### Mean glomerular volume assessment

The 2-profile method was applied to estimate the mean glomerular volume as described previously (Najafian, Basgen & Mauer, 2002). Briefly, at a thickness of 5 µm kidney samples were sectioned stained with periodic acid–Schiff (PAS) (*n* = 6). Two sections were made on parallel slides at 20-µm intervals. Then 10 individual glomeruli were randomly selected for imaging. The glomerular tuft was digitally traced in each captured image, and the areas were calculated using imaging software (NIS-Elements D 3.22; Nikon Instruments Inc., Melville, NY). Based on the areas of the two sections, the mean glomerular volume was calculated.

### Evaluation of glomerulosclerosis

Kidney sections (*n* = 6) stained with PAS were used for glomerulosclerosis demonstration. The degrees of glomerular damage were assessed semi-quantitatively using a Nikon Eclipse E600 optical microscope according to scoring method in randomly selected 100 glomeruli per section as follows: grade 0, normal glomeruli; grade 1, sclerotic area up to 25% (minimal sclerosis); grade 2, sclerotic area 25–50% (moderate sclerosis); grade 3, sclerotic area 50–75% (moderate-severe sclerosis); grade 4, sclerotic area 75–100% (severe sclerosis). The glomerulosclerotic index (GSI) was calculated using the following formula: GSI = (1 × *n*1) + (2 × *n*2) + (3 × *n*3) + (4 × *n*4)/*n*0 + *n*1 + *n*2 + *n*3 + *n*4, where *nx* is the number of glomeruli in each grade of glomerulosclerosis (Saito et al., 1987).

### Statistical analysis

The results of the current study are described as the mean and standard error (mean  ± SEM) (*n* = 6). One-way ANOVA was performed to test the significant differences among different groups. Newman-Keuls multiple comparison test was applied as a post hoc test. The data were considered significant when P ≤0.05. Statistical analyses were performed using Graph Pad Prism version 5.

## Results

### Effects of LCZ696 and valsartan on serum parameters

**Figure 1:**
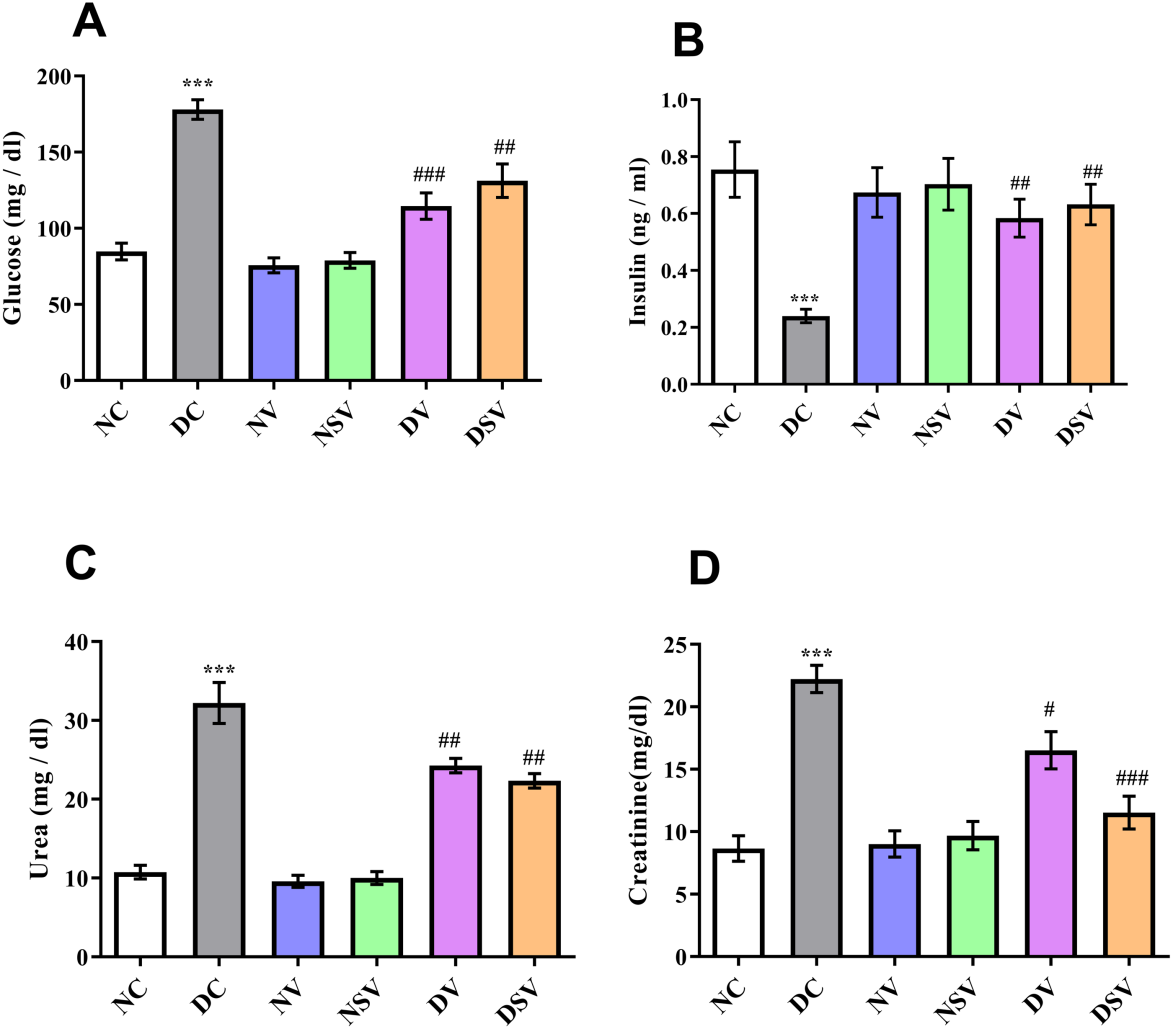
Effect of valsartan and LCZ696 on serum glucose (A), insulin (B), urea (C) and creatinine (D) levels in STZ-induced diabetic rats. Data are expressed as the mean ± SEM (*n* = 6 per group). Statistically significant difference: ^*^p < 0.001 versus NC group and ^#^*p* < 0.05, ^##^*p* < 0.01, ^###^*p* < 0.001 versus DC group. (ANOVA followed by Newman-Keuls multiple comparison test). NC, Normal Control; DC, Diabetic Control; NV, normal valsartan; NSV, normal sacubitril/valsartan; DV, diabetic valsartan; DSV, Diabetic sacubitril/valsartan.

Figure 1 shows the serum glucose, insulin, urea and creatinine levels in diabetic and treated groups. There was a significantly higher level (*P* < 0.001; DC vs. NC) of glucose in DC group with the mean value of 178 ± 6.4 mg/dl in comparison to NC group 84.7 ± 5.5 mg/dl. After 6 weeks of treatments with valsartan and LCZ696 in the diabetic rats, significant reduction (*P* < 0.001; DV vs. DC and DSV vs. DC) in serum glucose levels were seen with the mean serum glucose values were 114.6 ± 8.6 mg/dl in DV group and 131.2 ± 11 mg/dl in DSV. A three folds decrease (*P* < 0.001) in serum insulin levels (0.24 ± 0.02 ng/ml) were seen in the DC when compared to NC (0.75 ± 0.1 ng/ml). Furthermore, in diabetic groups of animals with time course therapy of valsartan and LCZ696, a significant restoration of insulin levels were observed and amounting to 0.58 ± 0.06 and 0.63 ± 0.07 ng/ml (*P* < 0.01–0.001 vs. DC). Urea level observed in the DC group was 32.2 ± 2.6 mg/dl and after 6 weeks of treatments levels noticed in DV were 24.2 ± 0.9 mg/dl (*P* < 0.001 compared to DC) and in DSV 22.3 ± 0.9 mg/dl (*P* < 0.001 compared to DC). Finally the serum level of creatinine observed in treated DV and DSV were 16.52 ± 1.4 mg/dl and 11.54 ± 1.3 mg/dl in comparison to DC 22.2 ± 1.0 mg/dl (*P* < 0.01–0.001 vs. DC).

### Effects of LCZ696 and valsartan on serum inflammatory cytokines

**Figure 2:**
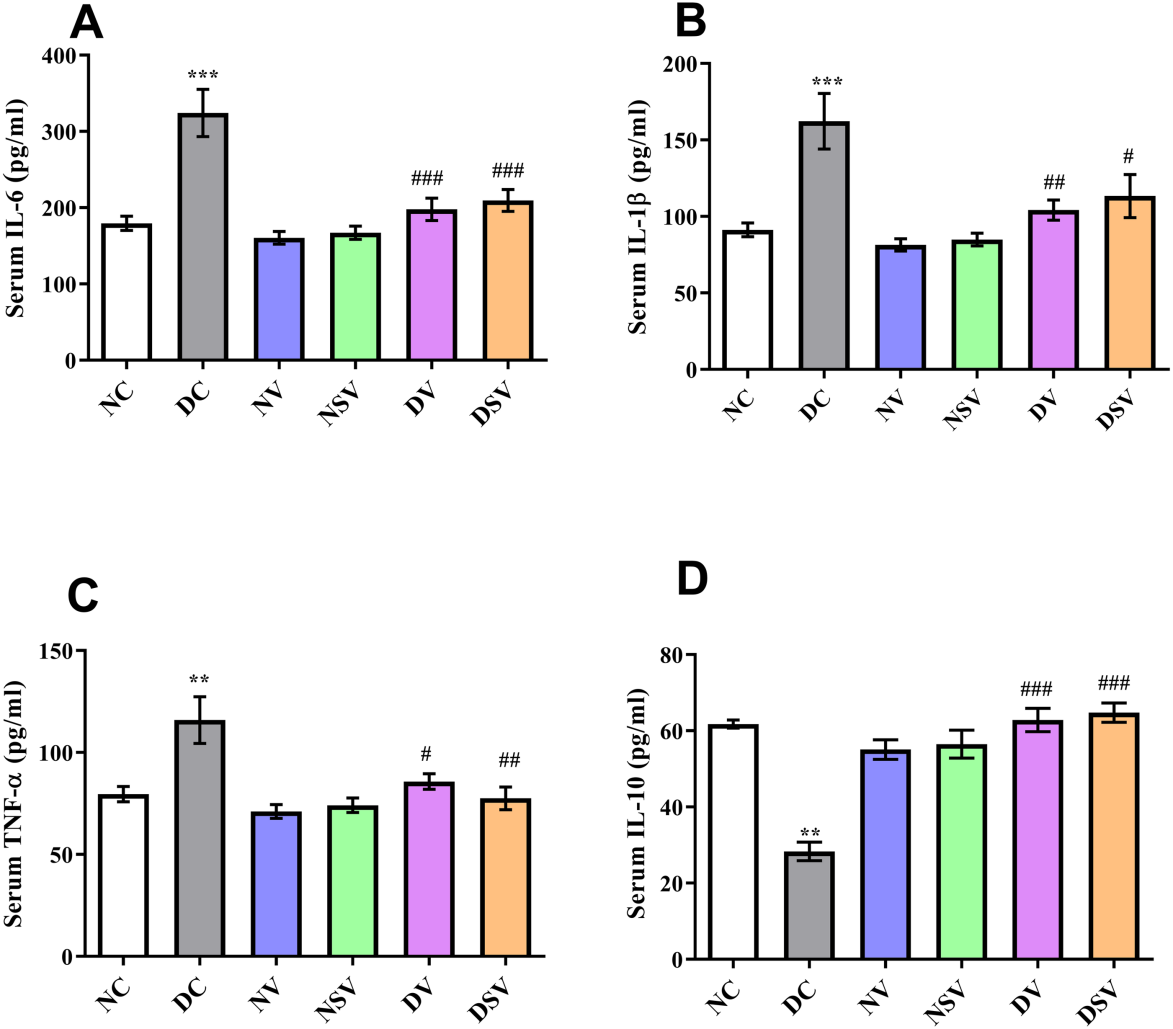
Effect of valsartan and LCZ696 on serum pro-inflammatory cytokines including interleukin-6 (IL-6) (A), interleukin-1β (IL-1β) (B), tumor necrosis factor-α (TNF-α) (C) and interleukin-10 (IL-10) (D) in STZ-induced diabetic rats. Data are expressed as the mean ± SEM (*n* = 6 per group). Statistically significant difference: ^**^p < 0.01,^***^p < 0.001 versus NC group and ^#^*p* < 0.05, ^##^*p* < 0.01, ^###^*p* < 0.001 versus DC group. (ANOVA followed by Newman-Keuls multiple comparison test). NC, Normal Control; DC, Diabetic Control; NV, normal valsartan; NSV, normal sacubitril/valsartan; DV, diabetic valsartan; DSV, Diabetic sacubitril/valsartan.

The serum levels of pro-inflammatory cytokines (IL-1β, IL-6, TNF-α) and anti-inflammatory marker IL-10 were assessed by ELISA in control, diabetic and treated groups and presented in Fig. 2. In DC group, the level of IL-6 was found significantly higher (324.1 ± 31 pg/ml; *P* < 0.001) in comparison to normal control (179.6 ± 9.3 pg/ml) and treated groups (160.4 ± 8.3 pg/ml for NV, 167.3 ± 8.6 pg/ml for NSV, 198 ± 14.7 pg/ml for DV and 209.5 ± 14.3 pg/ml for DSV; *P* < 0.05 compared to DC group). In comparison to DC, there was 2-folds increase seen in case of IL-1β with the DC group and amounting to 162.3 ± 18.16 pg/ml, *P* < 0.001. Subsequent to 6- week’s treatments with valsartan and LCZ696 in the diabetic rats, the observed levels of IL-1β were found statistically significant (*P* < 0.001) in comparison to DC group and values were 104.2 ± 6.6 pg/ml for DV group and 113.3 ± 14.06 pg/ml for DSV treated group. Moreover, the level of TNF-*α* were significantly reduced in STZ induced diabetes treated groups with valsartan (85.74 ± 3.85 pg/ml; *P* < 0.01 vs. DC group) and LCZ696 (77.52 ± 5.5 pg/ml; *P* < 0.001 vs. DC group).

On the other hand, in DC group ELISA assay revealed the substantial reduction (*P* < 0.001) in the serum level of anti-inflammatory marker IL-10 in comparison to normal control group (DC: 28.3 ± 2.44 pg/ml vs. NC: 61.67 ± 1.09 pg/ml). Interestingly, both LCZ696 or valsartan treated animals exhibited a pronounced restoration (*P* < 0.05) in the serum level of IL-10 compared with DC group and therefore an-indication of existence of anti-inflammatory properties of the used drugs.

### Effects of LCZ696 and valsartan on renal inflammatory biomarkers

**Figure 3:**
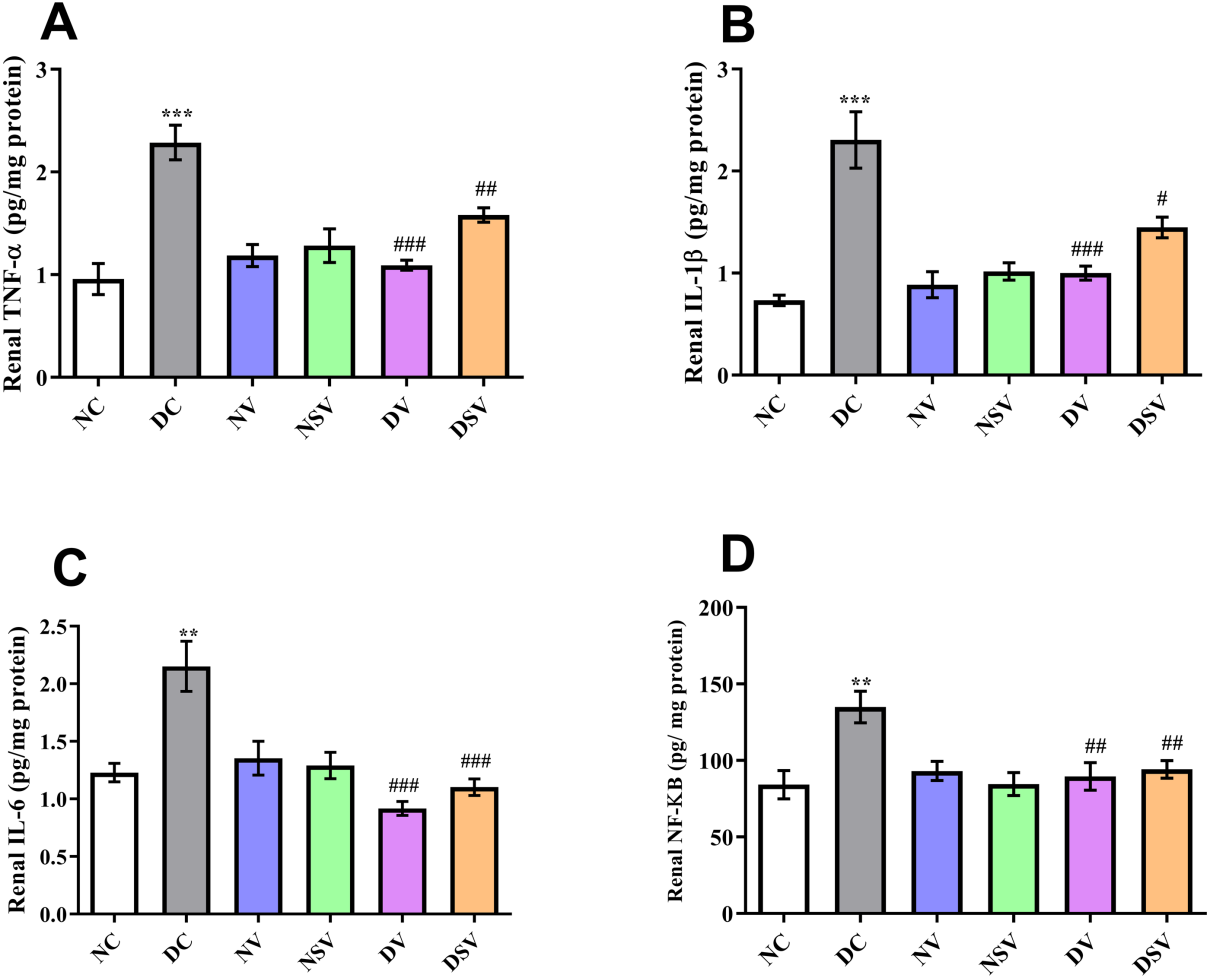
Effect of valsartan and LCZ696 on renal inflammatory bio-markers including tumor necrosis factor-α (TNF-α) (A), interleukin-1β (IL-1β) (B), interleukin-6 (IL-6) (C) and nuclear factor kappa-B (NF-κB) (D) in STZ-induced diabetic rats (*n*= 6 per group). Statistically significant difference: ^**^p < 0.01, ^***^p < 0.001 versus NC group and ^#^*p* < 0.05, ^##^*p* < 0.01, ^###^*p* < 0.001 versus DC group. (ANOVA followed by Newman-Keuls multiple comparison test). NC, Normal Control; DC, Diabetic Control; NV, normal valsartan; NSV, normal sacubitril/valsartan; DV, diabetic valsartan; DSV, Diabetic sacubitril/valsartan.

The levels of inflammatory bio-markers such as TNF-α, IL-1β, IL-6 and NF-kB in the tissue homogenate were assessed by ELISA in control, diabetic and treated groups and presented in Fig. 3. When the levels of bio-markers were statistically compared between DC and NC group, a significant difference was observed in TNF-α (DC: 2.3 ± 0.17 pg/mg of protein vs. NC: 0.95 ± 0.15 pg/mg of protein; *P* < 0.001), IL-1β (DC: 2.3 ± 0.28 pg/mg of protein vs. NC: 0.73 ± 0.05 pg/mg of protein; *P* < 0.001), IL-6 (DC: 2.15 ± 0.21 pg/mg of protein vs. NC: 1.23 ± 0.08 pg/mg of protein; *P* < 0.001) and NF-kB (DC: 135 ± 10.38 pg/mg of protein vs. NC: 84.18 ± 9.19 pg/mg of protein; *P* < 0.01). In all diabetic groups treated with valsartan and LCZ696 for the duration of six weeks, the levels of bio-markers were reduced significantly (*P* < 0.001) in comparison to DC group.

### Effects of LCZ696 and valsartan on oxidative stress biomarkers

**Figure 4:**
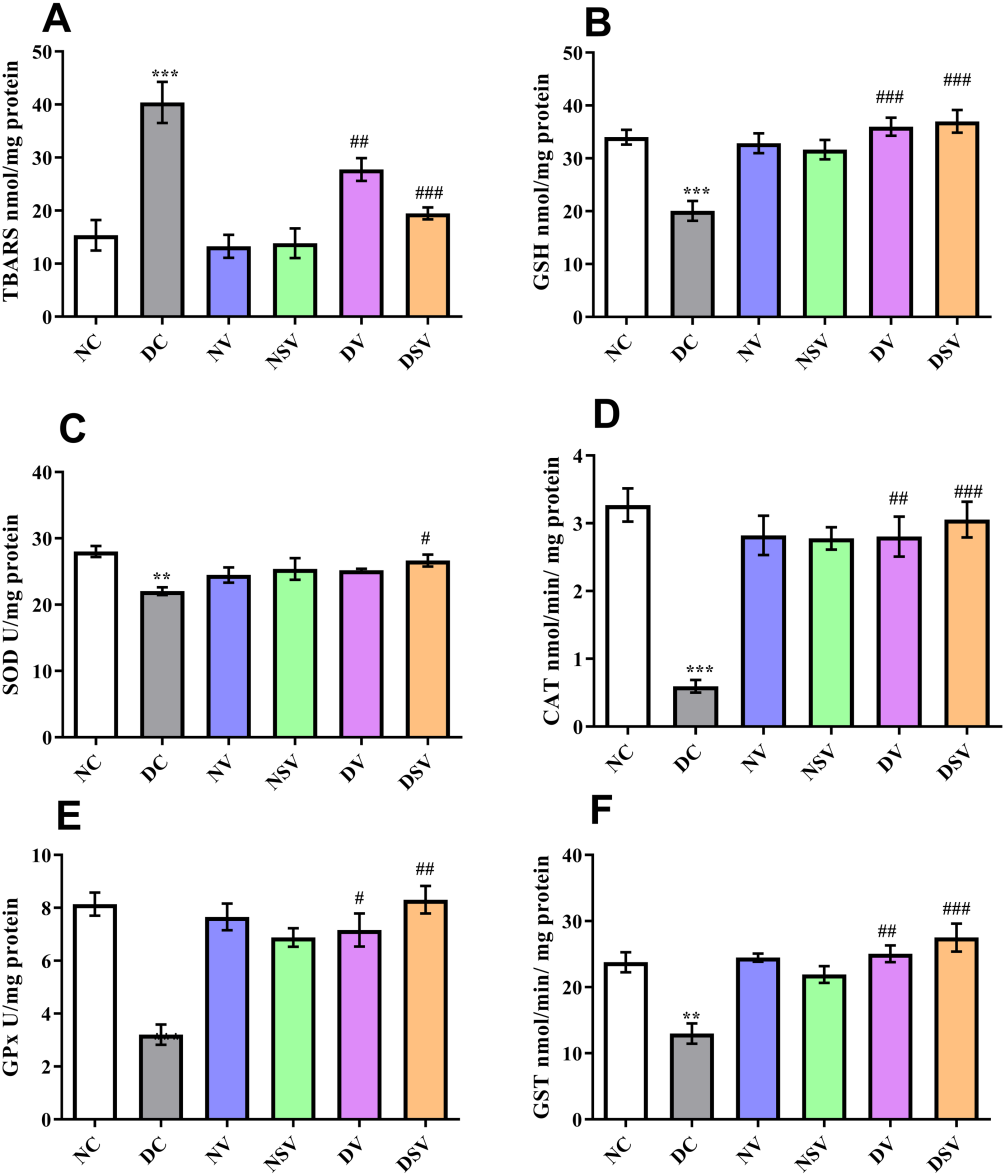
Effect of valsartan and LCZ696 on thiobarbituric acid reaction substances (TBARs) (A), glutathione (GSH) (B), superoxide dismutase (SOD) (C), catalase (CAT) (D), glutathione peroxidase (GPx) (E) and glutathione-S-transferase (GST) (F) in diabetic rats. Statistically significant difference: ^**^p < 0.01, ^***^p < 0.001 versus NC group and ^#^*p* < 0.05, ^##^*p* < 0.01, ^###^*p* < 0.001 versus DC group (*n* = 6 per group). (ANOVA followed by Newman-Keuls multiple comparison test). NC, Normal Control; DC, Diabetic Control; NV, normal valsartan; NSV, normal sacubitril/valsartan; DV, diabetic valsartan; DSV, Diabetic sacubitril/valsartan.

As demonstrated in Fig. 4, the oxidative stress biomarkers including TBARs, GSH, CAT, SOD, GPx and GST were estimated in renal tissue of control, diabetic and treated groups (valsartan and LCZ696 over six weeks). The levels of renal TBARs in the DC group of rats were significantly (*p* < 0.001) increased and found to be 40.40 ± 3.88 nmol/mg protein as compared to NC group (15.36 ± 2.86 nmol/mg protein). Furthermore , with the other bio-markers of oxidative stress pertaining to DC and NC groups, significant difference was detected in GSH (DC: 20.05 ± 1.88 nmol/mg protein vs. NC: 34 ± 1.41 nmol/mg protein; *P* < 0.001), SOD (DC: 22.05 ± 0.58 U/mg protein vs. NC: 28.01 ± 0.83 U/mg protein; *P* < 0.01), CAT (DC: 0.59 ± 0.09 mmol/min/mg protein vs. NC: 3.27 ± 0.24 mmol/min/mg protein; *P* < 0.001), GPx (DC: 3.2 ± 0.38 U/mg protein vs. NC: 8.14 ± 0.43 U/mg protein; *P* < 0.001) and GST (DC: 12.99 ± 1.53 mmol/min/mg protein vs. NC: 23.78 ± 1.51 mmol/min/mg protein; *P* < 0.001).

Subsequent to 6-week’s treatments with valsartan and LCZ696 in the diabetic rats, the observed level of TBARs were found statistically significant (*P* < 0.01–0.001) in comparison to DC group and values were 27.76 ± 2.135 nmol/mg protein for DV group and 19.47 ± 1.12 nmol/mg protein for DSV treated group. The level of GSH was significantly increased in STZ induced diabetes treated groups with valsartan (35.98 ± 1.71 nmol/mg protein; *P* < 0.01 vs. DC group) and LCZ696 (37 ± 2.15 nmol/mg protein; *P* < 0.001 vs. DC group). With the exception of SOD where a level of significance was observed with only DSV treated group (*P* < 0.05 vs. DC group), rest of three oxidative stress parameters in treated groups such as CAT, GPx and GST have shown a significant increase in respective anti-oxidant enzymatic levels in comparison to diabetes group (*P* < 0.001 vs. DC group). Interestingly, the time course therapy of only valsartan and LCZ696 in normal group of rats have not altered the levels of defensive anti-oxidant enzyme and found comparable to NC groups (Fig. 4).

### Effect of LCZ696 and valsartan on renal cortex histology

**Figure 5:**
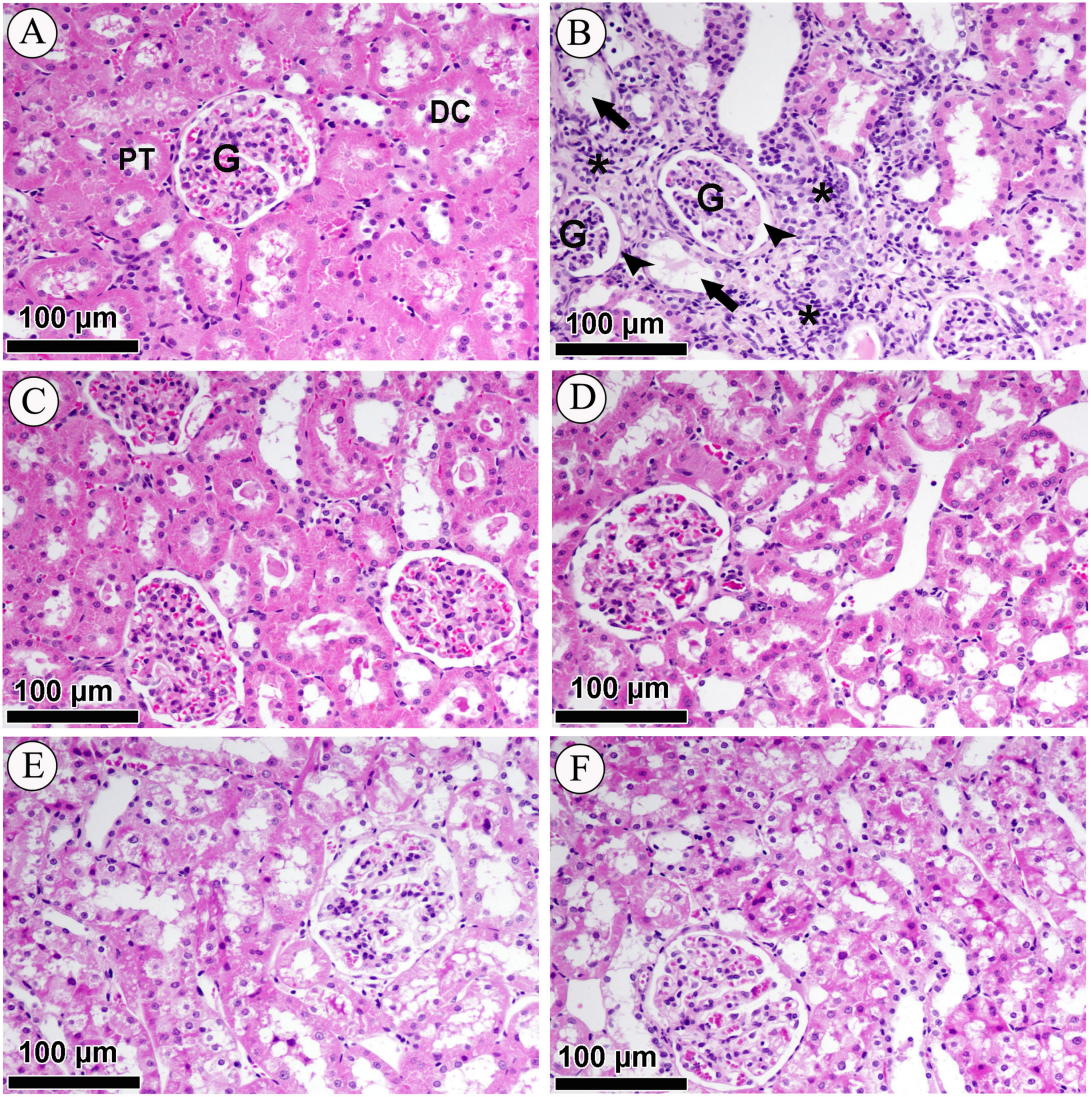
Effect of valsartan and LCZ696 on STZ-induced pathological changes in the kidney as indicated with H&E staining. (A) Section from the renal cortex of the NC group reveals the normal appearance of the proximal convoluted tubules (PT), distal convoluted tubules (DT), glomerulus (G) and Bowman’s capsule. (B) DC group indicates renal cortex with intense diffused interstitial inflammation (asterisk), vacuolarization of renal cells (arrow), necrosis, tubular degeneration and fragmented glomeruli with an increase in Bowman’s space (head arrows). (C) Normal rats-treated with valsartan (NV). (D) Normal rats treated with LCZ696 (NSV). (E) Diabetic rats-treated with valsartan (DV), (F) diabetic rats-treated with LCZ696 showed remarkably reduced renal injury with limited necrosis and inflammatory cells infiltration.

Photomicrographs of H&E-stained renal sections of control, diabetic and treated groups are shown in Fig. 5. The sub-section of Fig. 5A has demonstrated the histology of renal cortex and revealed normal structure of the proximal convoluted tubules, distal convoluted tubules, Bowman’s capsule, and glomeruli in the NC group. Figure 5B belongs to DC group and indicating that renal cortex is severely compromised with intense diffused interstitial inflammation, vacuolarization of renal cells, necrosis, tubular degeneration and fragmented glomeruli with an increase in Bowman’s space. Figures 5C & 5D is affiliated with NV (valsartan) and NSV (LCZ696) groups and stained sections showed sections didn’t show any alterations in tubular or glomerular tissues. Lastly in Figs. 5E & 5F H&E staining revealed that treatment of diabetic rats with valsartan and LCZ696 remarkably reduced renal injury with limited necrosis and inflammatory cells infiltration.

### Effects of LCZ696 and valsartan treatment on glomerular hypertrophy in diabetic rats

**Figure 6:**
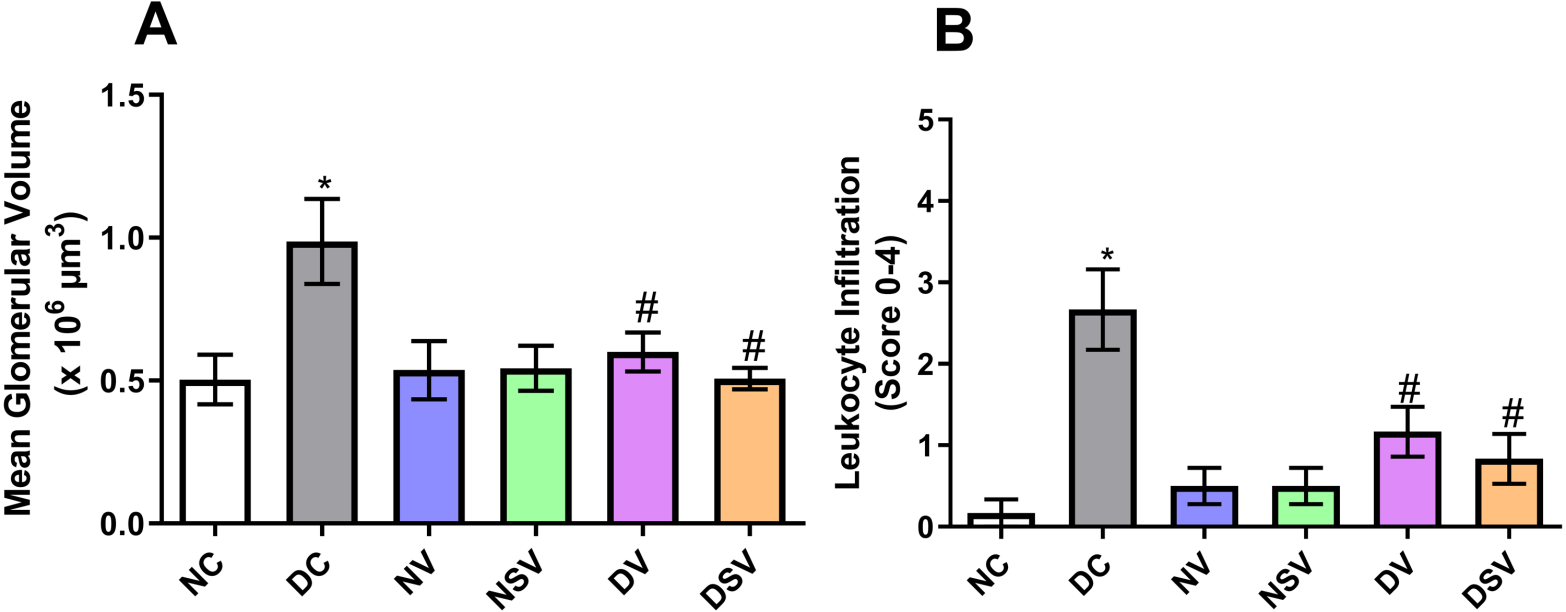
Effect of valsartan and LCZ696 on renal glomerular volume (A) and leukocyte infiltration score (B) in STZ-induced diabetic rats. Statistically significant difference: ^*^p < 0.05 versus NC group, ^#^*p* < 0.05 versus DC group. (ANOVA followed by Newman-Keuls multiple comparison test). NC, Normal Control; DC, Diabetic Control; NV, normal valsartan; NSV, normal sacubitril/valsartan; DV, diabetic valsartan; DSV, Diabetic sacubitril/valsartan.

The glomerular volume values and leukocyte infiltration (score) are shown in Fig. 6. Our results showed that the mean glomerular volume was significantly greater (2 fold) in DC group than in NC group (NC, 0.5 ± 0.08 106 µm3 vs. DC, 0.99 ± 0. 15 × 106 µm3; *p* = 0.048). Treatment of diabetic animals with valsartan (DV group) inhibited glomerular hypertrophy (−38%) in comparison with DC group. Interestingly, LCZ696 treatment (DSV group) significantly inhibited the increase in the mean glomerular volume compared to untreated DC group. The inflammatory cells were evaluated by scoring the amount of infiltrates on a scale of 0–4 in H&E stained sections (Fig. 6B). Our results showed that leukocyte infiltration in DC group was significantly higher (15.8 fold) than that of NC animals. Leukocyte infiltration levels in diabetic rats treated with valsartan (DV group) and LCZ696 (DSV group) were significantly lower (−57% and −68% respectively) than that of DC animals.

### Effect of LCZ696 and valsartan on glomerulosclerosis in diabetic rats

**Figure 7:**
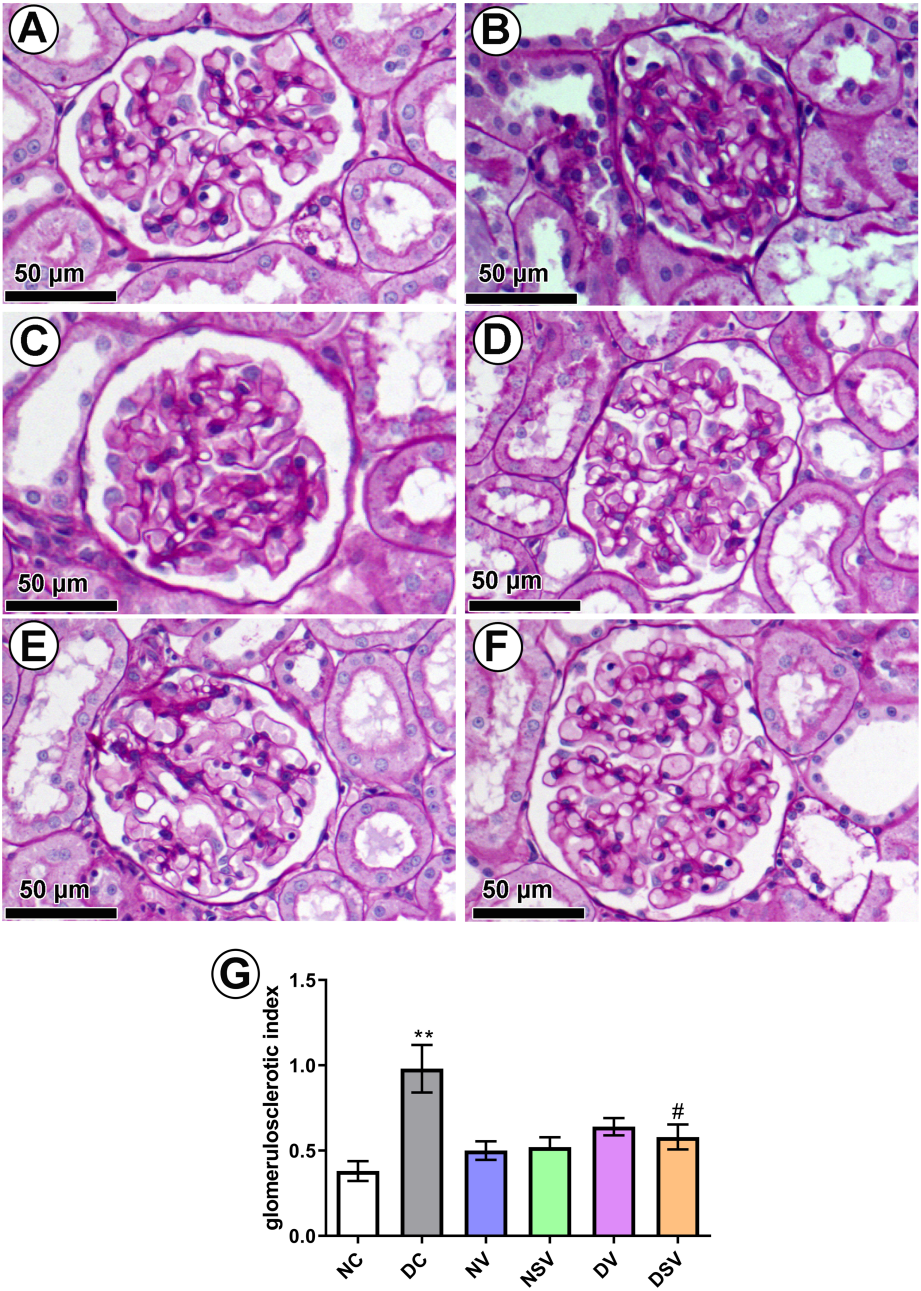
Effect of valsartan and LCZ696 on glomerulosclerosis in diabetic rats. Representative photomicrographs of glomeruli stained by periodic acid-Schiff (PAS, scale 50 µm) from control (A), DC (B), normal valsartan (NV) (C), normal LCZ696 (NSV) (D), diabetic valsartan (DV) (E), and diabetic LCZ696 (DSV) (F)—treated rats. (G) Quantification of the glomerular matrix score . Statistically significant difference: ^**^p < 0.01 versus NC group, ^#^*p* < 0.05 versus DC group. (ANOVA followed by Newman-Keuls multiple comparison test). NC, Normal Control; DC, Diabetic Control; NV, normal valsartan; NSV, normal sacubitril/valsartan; DV, diabetic valsartan; DSV, Diabetic sacubitril/valsartan.

PAS-stained sections (Figs. 7A–7F) were used to quantify the percentage of glomerulosclerosis in DC group. The accumulated data from six individual rats from each group are shown in Fig. 7-G. Glomerulosclerosis was significantly increased in the DC group in comparison with the NC group (NC, 0.38 ± 0.058 vs. DC, 0.98 ± 0.13; *p* = 0.004). Interestingly, valsartan (DV) and LCZ696 (DSV) treatment have effectively prevented the rise in glomerulosclerosis in animals with diabetes.

## Discussion

In the present study, we demonstrated that a definite time course therapy (6 weeks) with LCZ696 in streptozotocin induced diabetic rats resulted in normalization of serum glucose, insulin, urea and creatinine levels. Furthermore, serum of treated diabetic rats with LCZ696 exhibited a decrease in inflammatory markers (TNF-*α*, IL-1β, IL-6) and augmentation in anti-inflammatory (IL-10) cytokines levels and hence an indication of therapeutic role of LCZ696. To justify the protective role of LCZ696, tissue levels of inflammatory TNF-α, IL-1β, IL-6 and NF-kB were assessed and found to be decreased after 6 weeks of LCZ696 treatment in diabetic animals. Our ELISA studies relate to the measurement of anti-oxidants enzymes levels in kidney homogenate strongly supports the restoration of enzymatic activities of superoxide dismutase (SOD), catalase (CAT), glutathione peroxidase (GPx) and glutathione-S-transferase (GST) by a angiotensin receptor-neprilysin inhibitors i.e., LCZ696. In the histological sections of the kidney, prevention of renal injury was observed with limited necrosis and inflammatory cells infiltration.

Many scientific studies have linked DN with oxidative stress, inflammation and glomerulosclerosis (Mou et al., 2019; Saito et al., 1987). Approaches to mitigate these pathological processes can therefore be effective in limiting the progression of DN (Pickering et al., 2018). It has been reported in several studies that persistent hyperglycemia provokes the generation of reactive oxygen species (ROS) which contribute to oxidative damage to the macromolecules (lipids, carbohydrates, proteins, and nucleic acids) and eventually tissue injury. Persistent hyperglycemia causes (ROS) to be produced in cells which lead to oxidative damage to macromolecules (lipids, carbohydrates, proteins and nucleic acids) and ultimately to tissue injury (Sifuentes-Franco et al., 2018). Scientific literature have also provided evidence that production of proinflammatory cytokines contribute to a variety of pathological pathways which are linked to the development of DN (Donate-Correa et al., 2015). Similarly, this study supports certain existing findings as well as introduces other markers of elevated systemic and tissue oxidative injuries and inflammation. Moreover, DN has been confirmed in this study by altered creatinine and urea levels as markers of renal dysfunction, accompanied by structural changes including glomerular mesangial expansion and glomerulosclerosis. Along with these markers, there were elevations in the levels of the proinflammatory cytokines and lipid peroxidation as well as deficiency in the endogenous antioxidant molecules and enzyme activities in the diabetic animals.

Here, we observed that both LCZ696 and valsartan treatments could limit oxidative damage, lipid peroxidation and inflammation. Moreover, both therapies protected renal functions and prevented incidence of glomerulosclerosis in diabetic animals. Recently, it has been reported that simultaneous neprilysin inhibition and renin-angiotensin system modulations prevented diabetic nephropathy and our results confirmed the same (Malek et al., 2019). The therapeutic potential of LCZ696 has been reported by Jing et al. (2017) who found that LCZ696 more effective than valsartan therapy in delaying the progression of kidney disease in 5/6 nephroctomy model. Notably, evidence from animal and clinical studies suggested that RAS blockade was a well effective strategy in preventing renal injury through modulation of oxidative stress and inflammation levels (Aminzadeh, Sato & Vaziri, 2012). Additionally, this improvement in renal structure and functions after LCZ696 therapy may be based on augmentation of natriuretic peptides (NPs) mediated by inhibition of neprilysin. Many clinical and preclinical studies have shown that increased levels of vasoactive peptides including NPs prevent renal injury and delay the development of renal diseases (Morikawa et al., 2009; Nojiri et al., 2015). The antioxidant and anti-inflammatory properties of NPs may have these beneficial effect on the kidneys (Judge et al., 2015).

NF-kB is a crucial transcription factor in immune responses and in the expression of specific DN cytokines (Yi et al., 2017). The results of the current study show that LCZ696 therapy inhibits the elevated levels of NF-κB in diabetic animals. The results of this study are consistent with those of other previous studies (Jing et al., 2017), who found LCZ696 was more effective in delaying the progression of kidney disease than valsartan therapy alone. Unlike, the current study in which both drugs performed very similarly and this may be due to the difference in the two models, doses of both drugs and the duration of the experiment. Likewise, LCZ696 treatment for 8 weeks inhibited NF-κB activation and consequently restricted cardiovascular and renal functional decline in the model of chronic kidney disease induced by 5/6 nephrectomy in male rats (Suematsu et al., 2018). In addition, it has been recently demonstrated that atrial natriuretic peptide (ANP) inhibits the activation of NF-κB pathway, thereby, reducing the production of ROS and cytokines release (Mezzasoma, Antognelli & Talesa, 2016).

Intriguingly, the present work shows that the treatment with LCZ696 can minimize the pathological manifestations of the DN as shown by a significant reduction of leukocyte infiltration, mean glomerular volume and glomerulosclerosis. Similarly, Habibi et al. (2019) have demonstrated that glomerular and tubular injury was attenuated by the combination of a neprilysin inhibitor (sacubitril) and angiotensin-II receptor blocker (valsartan) in the Zucker Obese rat. This improvement in renal structure might be attributed to the ability of LCZ696 in reducing oxidative stress and inflammation. Although similarities exist between the above studies and the current research, there is significant difference in the diabetic model that has been used to evaluate the therapeutic advantages of LCZ696. The efficacy of LCZ696 therapy was comparable in DN attenuation compared to valsartan alone, which could be attributed to the lack of specificity of these medications in the treatment of renal diseases or may be no synergistic effects of dual blockade of the angiotensin II receptor and neprilysin.

## Conclusions

Taken together, our data provide an evidence that LCZ696 has therapeutic potential to restrict DN progression by inhibiting oxidative stress, NF-kB mediated inflammation and glomerulosclerosis.

##  Supplemental Information

10.7717/peerj.9196/supp-1 Data S1 Raw data of serum, tissue and morphometic measurements

## Additional Information and Declarations

### Data Availability

The following information was supplied regarding data availability: The raw measurements are available in Supplemental File.
